# The protein level and transcription activity of activating transcription factor 1 is regulated by prolyl isomerase Pin1 in nasopharyngeal carcinoma progression

**DOI:** 10.1038/cddis.2016.349

**Published:** 2016-12-29

**Authors:** Guo-Liang Huang, Dan Liao, Hua Chen, Yan Lu, Liyong Chen, Huahui Li, Binbin Li, Weilong Liu, Caiguo Ye, Tong Li, Zhu Zhu, Jian Wang, Takafumi Uchida, Ying Zou, Zigang Dong, Zhiwei He

**Affiliations:** 1 China-American Cancer Research Institute, Dongguan Scientific Research Center, Guangdong Medical University, Dongguan, China; 2 Key Laboratory for Epigenetics of Dongguan City, Key Laboratory for Medical Molecular Diagnostics of Guangdong Province, Dongguan, China; 3 Department of Gynaecology and Obstetrics, Dongguan Third People's Hospital, Affiliated Dongguan Shilong People's Hospital of Southern Medical University, Dongguan, China; 4 Research Institute of Clinical Medicine, The First People's Hospital of Shunde Affiliate to Southern Medical University, Foshan, China; 5 Experimental Animal Center, Shenzhen Third People's Hospital, Shenzhen, China; 6 Department of Molecular Cell Biology, Graduate School of Agricultural Science, Tohoku University, Sendai, Miyagi, Japan; 7 The Hormel Institute, University of Minnesota, Austin, MN, USA

## Abstract

The function of activating transcription factor 1 (ATF1) and the mechanism about why ATF1 was over-phosphorylated in nasopharyngeal carcinoma (NPC) progression is completely undiscovered. In this study, a series of experiments both *in vitro* and *in vivo* were used to characterize a promotive function of ATF1 in NPC tumorigenesis and identify prolyl isomerase Pin1 as a novel regulator of ATF1 at post-transcription. First, we found that overexpression of ATF1 promoted colony formation in NPC. However, the high protein level of ATF1 in NPC was not resulted from high mRNA level. Then, a direct interaction between Pin1 and ATF1 at Thr184 was demonstrated using mammalian two-hybrid assay and coimmunoprecipitation. Cycloheximide (CHX) treatment indicated Pin1 stabilized the expression of ATF1 at post-transcription level. We confirmed that Pin1 upregulated ATF1 transcriptional activity of Bcl-2 using luciferase reporter assay, quantitative RT-PCR and western blot. Furthermore, the newly identified phosphorylation of ATF1 at Thr184 was suggested to have an important role in ATF1 function of transcription and tumor promotion. Finally, high expression of Pin1 in NPC tissue was found to be positively correlated with ATF1. The ATF1 promoted NPC tumorigenesis was regulated by Pin1 both *in vitro* and *in vivo.* All these findings clearly state that Pin1 is a novel regulator of ATF1 at Thr184 and thereby enhances ATF1 transcription activity and tumorigenesis promotive function in NPC.

Activating transcription factor 1 (ATF1) is a member of the ATF/CREB family of transcription factors (TFs), specifically interacting with the consensus ATF/CRE site ‘TGACGTCA'.^1^ CREB and ATF1 are required for t-Darpp upregulating Bcl-2 levels in resistance to ceramide-induced apoptosis in gastric cancer.^2^ Furthermore, expression of single chain antibody fragment anti-ATF1 in melanoma cells is found to suppresses the ATF1 tumorigenicity and metastatic potential in nude mice.^3^ Nasopharyngeal carcinoma (NPC), which is the most common cancer originating in the nasopharynx, has a high incidence in Southern China and Southeast Asia.^4, 5, 6^ In NPC, overexpression and over-phosphorylation of ATF1 is found to be associated with clinical stage.^7^ However, the function of ATF1 overexpression and the mechanism about why ATF1 was over-phosphorylated in NPC progression is completely undiscovered.

The mainly reported post-transcription regulatory mechanism of ATF1 is phosphorylation.^8^ Phosphorylation of ATF1 at Ser63 enforce its binding to the ATF/CRE site.^9^ The phosphorylation of ATF1 at Ser63 is induced by several serine/threonine kinases in various cellular background or stress.^10, 11, 12, 13^ By controlling proline-directed phosphorylation, peptidyl-prolyl isomerase Pin1 represents a novel regulatory mechanism of many TFs, such as p53, c-Jun, c-Fos, NF-*κ*B and *β*-catenin.^14, 15, 16, 17, 18, 19^ It is interesting to investigate whether the phosphorylation of ATF1 would be modulated by Pin1.

Pin1 is found to be a key regulator in cell transformation and oncogenesis.^20^ Pin1 contributes to the development of different cancers by targeting various phosphoproteins, such as hepatitis B virus X-protein in liver cancer ^21^ and estrogen receptor-alpha in breast cancer.^22^ In this study, we showed the role of ATF1 in NPC tumorigenesis *in vitro and in vivo*; clarified the protein level and transcriptional activity of ATF1 was regulated by Pin1; identified the new phosphorylated site at ATF1 Thr184 and its role in ATF1 function; and demonstrated Pin1 as a novel post-transcription regulator of ATF1 in NPC progression.

## Results

### High expression of ATF1 promotes cell tumorigenesis in NPC

**Figure 1:**
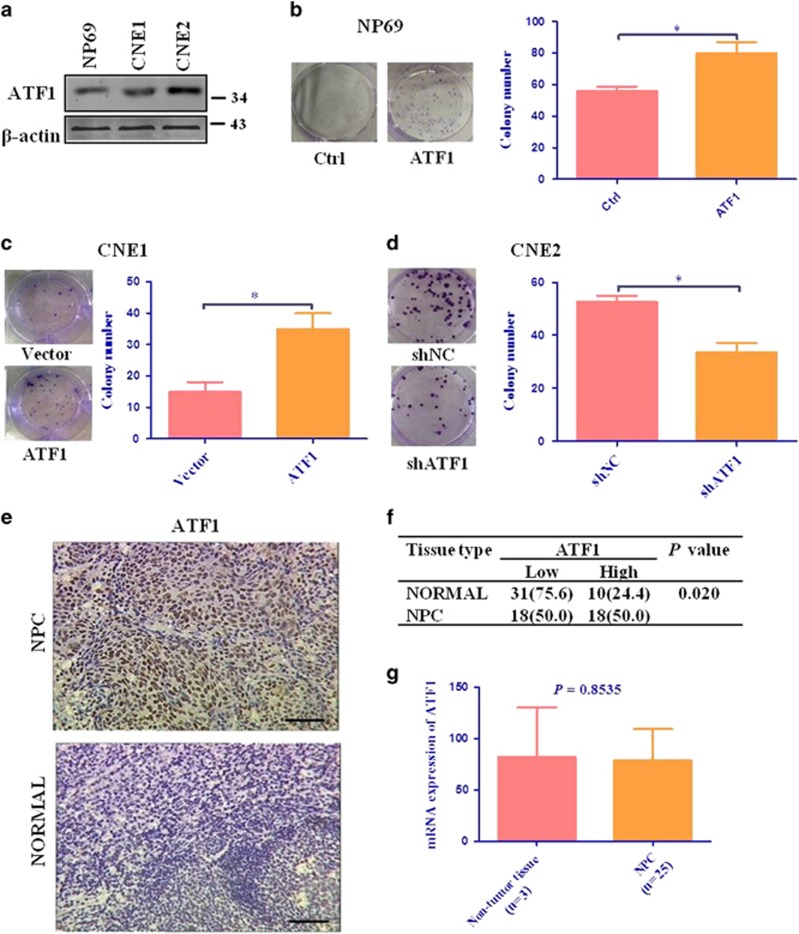
High expression of ATF1 promotes tumorigenesis in NPC. (**a**) Western blot assay to show the expression of ATF1 in various nasopharyngeal cells. (**b**) Colony formation assay showed the cell colony formation ability of NP69 cells with ATF1 overexpression. (**c**) Colony formation assay showed the cell colony formation ability of CNE1 cells with ATF1 overexpression. (**d**) Colony formation assay showed the cell colony formation ability of CNE2 cells with ATF1 knockdown. (**e**) Representative IHC staining of pATF1-Ser63 in NPC tissues and normal or reactive nasopharyngeal mucosal tissues. Scale bars, 50 *μ*m. (**f**) Correlation between nasopharyngeal tissues with pATF1-Ser63 levels. (**g**) The mRNA expression of ATF1 is not statistically different between NPC and non-tumor tissues in a GEO data set. 'shNC' stands for the negative control of shRNA. *P<0.05

The cell colony formation ability of ATF1 in NPC was assessed using colony formation assay with various alterations of ATF1 expression. Base on the expression levels of ATF1 in various nasopharyngeal cells (Figure 1a), in this study, we used CNE2 cells with high levels of ATF1 for knockdown experiments; NP69 cells with low levels of ATF1 for overexpression experiments; CNE1 with moderate levels of ATF1 for both knockdown and overexpression experiments whenever appropriate. The results indicated that overexpression of ATF1 increased the cell colony formation of CNE1 and NP69 cells, whereas knockdown of ATF1 decreased the cell colony formation ability of CNE2 cells (Figures 1b–d,Supplementary Figure S5A).

We next examined the levels of phosphorylated ATF1 at Ser63 (which represents the active state of ATF1) in NPC tissues using immunohistochemistry (IHC). The results indicated that the levels of pATF1-Ser63 was significantly higher in tumor tissues than adjacent/normal specimens (50.0% and 24.4%, respectively, *P*=0.020; Figures 1e and f). However, the mRNA expression of ATF1 was not higher in NPC compared with non-tumor tissues using the GEO data (Figure 1g). Rare mutation of ATF1 was found in NPC with the utility of another GEO data of 56 NPC (Supplementary Table S1). We infer that the highly phosphorylated level of ATF1 in NPC is caused by post-transcription regulation.

### Pin1 interacts with ATF1 at Thr184

**Figure 2:**
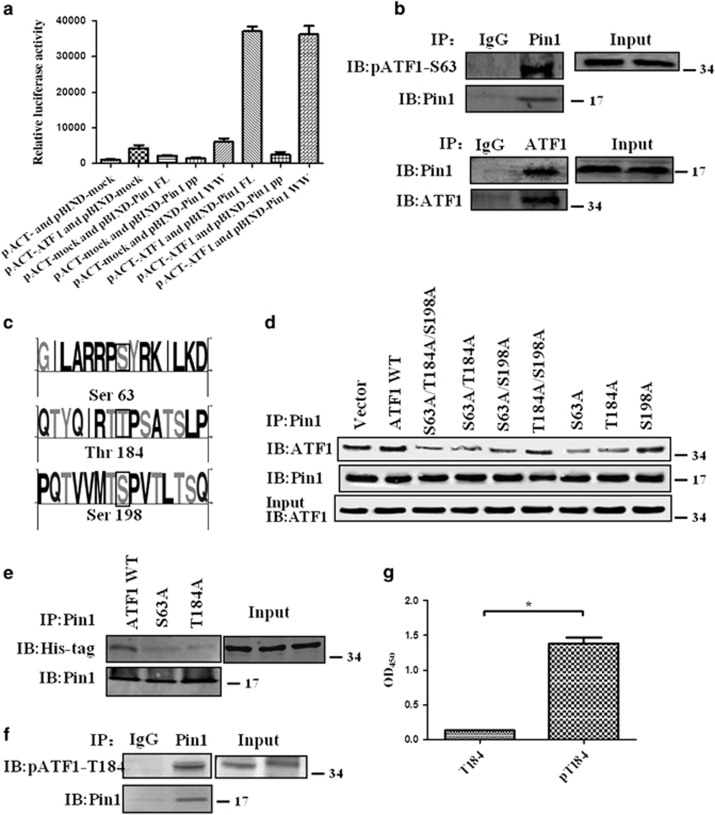
Pin1 interacts with ATF1 exogenously and endogenously in NPC, which is conferred by ATF1 phosphorylation sites at Thr184. (**a**) Mammalian two-hybrid assay showed the interaction between ATF1 and different domains of Pin1 using HEK-293 transfected with various plasmids for 24 h. (**b**) Coimmunoprecipitation experiments demonstrated a cellular interaction of Pin1 and ATF1 endogenously in NPC CNE2 cells. (**c**) Sequence logo showed the predicted phosphorylation sites at ATF1 that may affect the interaction between Pin1 and ATF1. (**d**) Coimmunoprecipitation experiments indicated that ATF1 Ser63Ala or Thr184Ala abolished the interaction between Pin1 and ATF1 after introducing recombinant ATF1 mutant proteins and Pin1 into CNE1 cells for 24 h. The remaining band of ATF1 was the endogenous interaction between Pin1 and ATF1 in the CNE1 cells as indicated in the first lane 'vector'. (**e**) Coimmunoprecipitation experiments with anti-His-tag antibody in IB to show ATF1 Ser63Ala or Thr184Ala abolished the interaction between Pin1 and ATF1 using CNE1 cells transfected with various plasmids for 24 hours. (**f**) Coimmunoprecipitation experiment demonstrated that phosphorylation of ATF1 at Thr184 was in the Pin1 immunoprecipitate endogenously in NPC CNE2 cells. (**g**) A modified ELISA assay demonstrated that the specific binding of Pin1 to phosphorylated T184 peptides *in vitro*. *P<0.05

We then examined whether there was a direct interaction between ATF1 and Pin1. In mammalian two-hybrid assay, the luciferase activity indicated a robust interaction between Pin1 and ATF1 and the interaction was mediated by WW domain, but not by PPIase domain, of Pin1 (Figure 2a). Coimmunoprecipitation experiments results showed that ATF1 was detected in the Pin1 immunoprecipitate and the presence of Pin1 specifically in the ATF1 immunoprecipitates of CNE2 cells (Figure 2b). The results showed a cellular interaction of Pin1 and ATF1 endogenously in NPC cells.

We used the NetPhos 2.0 to predict the phosphorylation sites at ATF1. The results indicated that Thr184 and Ser198 were predicted phosphorylation sites at ATF1 that matched the Pin1-binding sequence (Figure 2c). Therefore, we produced ATF1 mutants at these sites: Ser63Ala, Thr184Ala, Ser198Ala, Ser63Ala-Thr184Ala, Ser63Ala-Ser198Ala, Thr184Ala-Ser198Ala and Ser63Ala-Thr184Ala-Ser198Ala. Coimmunoprecipitation experiments indicated that ATF1 Ser63Ala or Thr184Ala abolished the interaction between Pin1 and ATF1 after introducing recombinant ATF1 mutant proteins and Pin1 into CNE1 cells (the remaining band of ATF1 in lane 'Ser63Ala' or 'Thr184Ala' was the endogenous interaction between Pin1 and ATF1 in the CNE1 cells as indicated in the first lane 'vector' Figures 2d and e). We infer that Thr184 is a new phosphorylation site at ATF1 and is a direct binding motif of Pin1. The phosphorylated Ser63 at ATF1 could modulate the protein tertiary structure of ATF1 and therefore affect its binding ability with Pin1. A further coimmunoprecipitation experiment demonstrated that phosphorylation of ATF1 at Thr184 was in the Pin1 immunoprecipitate (Figure 2f). A modified ELISA assay was performed to demonstrate the specific binding of Pin1 to phosphorylated T184 peptides *in vitro* (Figure 2g). The data showed high binding ability of Pin1 with the phosphorylated peptide pT184 but little binding with the non-phosphorylated peptide T184.

### Pin1 stabilizes the expression of ATF1 at post-transcription

**Figure 3:**
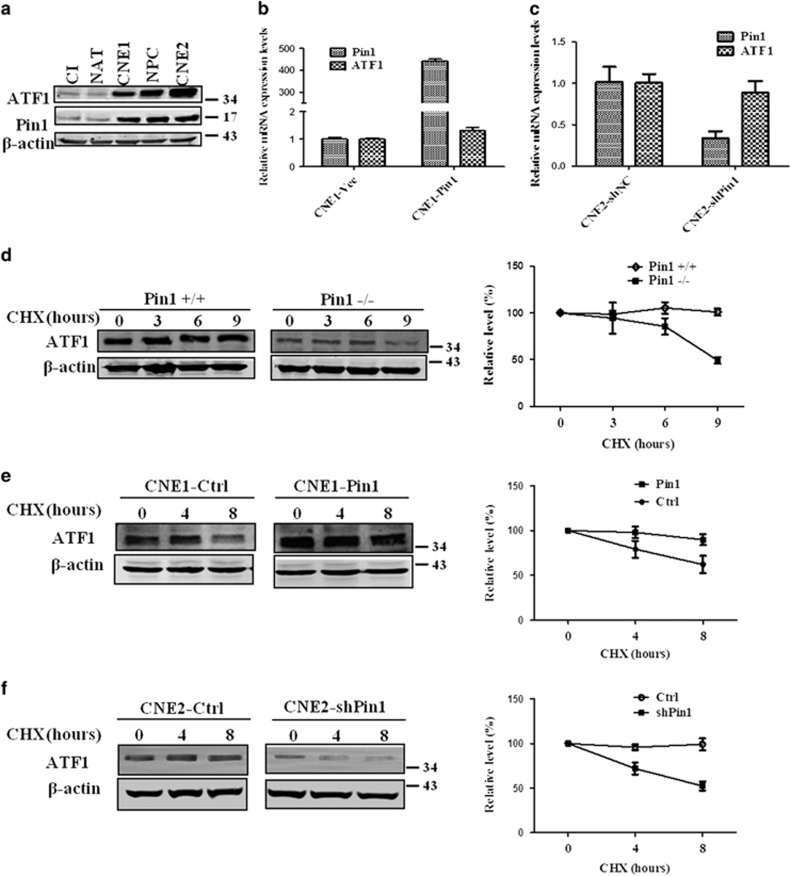
Pin1 stabilizes the expression of ATF1 in post-transcription level. (**a**) Western blotting displayed a positive correlation between Pin1 and ATF1 in a panel of NPC cell lines and NPC tissues. CI, chronic inflammation of nasopharynx tissue; NAT, normal adjacent tissue of NPC. (**b**) qRT-PCR assay showed the mRNA level of ATF1 was not regulated by upregulation of Pin1 using CNE1 cells transfected with Pin1 plasmid for 24 h. (**c**) qRT-PCR assay showed the mRNA level of ATF1 was not affected by downregulation of Pin1 using CNE2 cells transfected with shPin1 plasmid for 48 h. (**d**) Pin1^+/+^ and Pin1^−/−^ mouse embryonic fibroblasts cells were treated with 50 *μ*g/ml CHX for indicated durations followed by WB analysis. The quantitative data of ATF1 protein are represented in the right panel. (**e**) Stable Pin1 expression cells and control CNE1 cells were treated with 50 *μ*g/ml CHX for indicated durations followed by WB analysis. The quantitative data of ATF1 protein are represented in the right panel. (**f**) Stable Pin1 knockdown cells and control CNE2 cells were treated with 50 *μ*g/ml CHX for indicated durations followed by WB analysis. The quantitative data of ATF1 protein are represented in the right panel. 'shNC' stands for the negative control of shRNA

The protein expression of Pin1 was positively correlated with the expression of ATF1 in NPC cell lines and NPC tissues by western blot (Figure 3a). However, the mRNA level of ATF1 were not regulated by Pin1 neither by upregulation nor by downregulation (Figures 3b and c). To examine whether Pin1 affects ATF1 protein stability, we measured the half-life of ATF1 protein in several pairs of cells, including Pin1^+/+^ and Pin1^−/−^ embryonic fibroblasts cells, stable Pin1 expression cells and stable Pin1 knockdown cells, by means of cycloheximide (CHX) treatment (Figures 3d–f, Supplementary Figure S5B). The stability of ATF1 protein was significantly affected by Pin1 8–9 h post CHX treatment. These results indicated that Pin1 stabilized the protein expression of ATF1 at post-transcription level.

### Pin1 regulates ATF1 transcriptional activity

**Figure 4:**
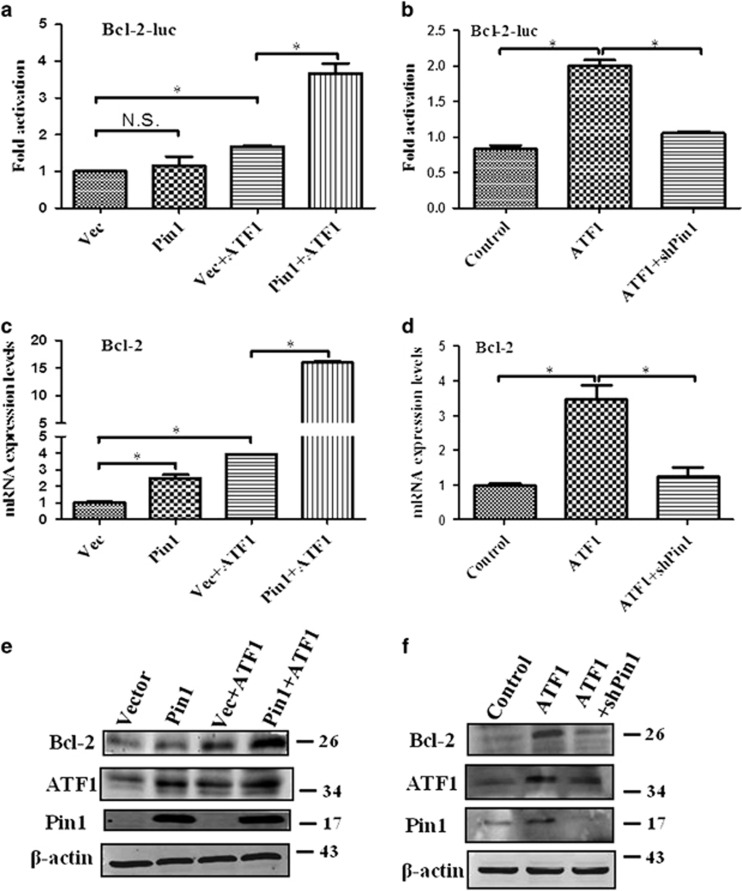
Pin1 regulates the transcriptional activity of ATF1 in NPC. (**a** and **b**) Luciferase assays showed Bcl-2 promoter activity regulated by various modifications of ATF1 and Pin1. (**c** and **d**) qRT-PCR assays showed Bcl-2 mRNA regulated by various modifications of ATF1 and Pin1. (**e** and **f**) Western blotting assays showed Bcl-2 protein regulated by various modifications of ATF1 and Pin1. CNE1 cells were transfected with overexpression plasmids for 24 h and with shPin1 plasmid for 48 h. In panels (**a**, **c** and **e**), vector was added in 'Vec+ATF1' to make this transfection system equal to 'Pin1+ATF1' (ATF1 plasmid was equal in the two transfection; whereas vector was used to replace Pin1 plasmid.). *P<0.05

As Bcl-2 was a transcriptional target of ATF1 that had an important role in tumorigenesis, the effect of Pin1 on the transactivation function of ATF1 was examined through the regulation of Bcl-2 by luciferase assay, qRT-PCR and western blot. Bcl-2 luciferase activity was significantly increased when ATF1 was co-transfected with Pin1 in NPC cells (Figure 4a). The mRNA and protein expression level of Bcl-2 in Pin1 or ATF1-overexpressing cells were higher than the level in control (Figures 4c and e). The mRNA and protein expression level of Bcl-2 in cells overexpression of both Pin1 and ATF1 was higher than the level in Pin1 or ATF1-overexpressing cells (Figures 4c and e).

Knockdown of Pin1 impaired the transcriptional activity of ATF1. The Bcl-2 luciferase reporter activity in CNE1 cells that simultaneous knockdown of Pin1 and overexpression of ATF1 was lower than that in ATF1-overexpressing cells (Figure 4b,Supplementary Figure S5C). The mRNA and protein expression level of Bcl-2 in CNE1 cells that simultaneous knockdown of Pin1 and overexpression of ATF1 were lower than that in ATF1-overexpressing cells (Figures 4d and f, and Supplementary Figure S5D and E). In other cancer cell lines HepG2 and A549, ATF1 transcriptional activation of Bcl-2 was also impaired by knockdown of Pin1 (Supplementary Figure S1).

### Mutation at Thr184 abrogates the biological function and transcriptional activity of ATF1

**Figure 5:**
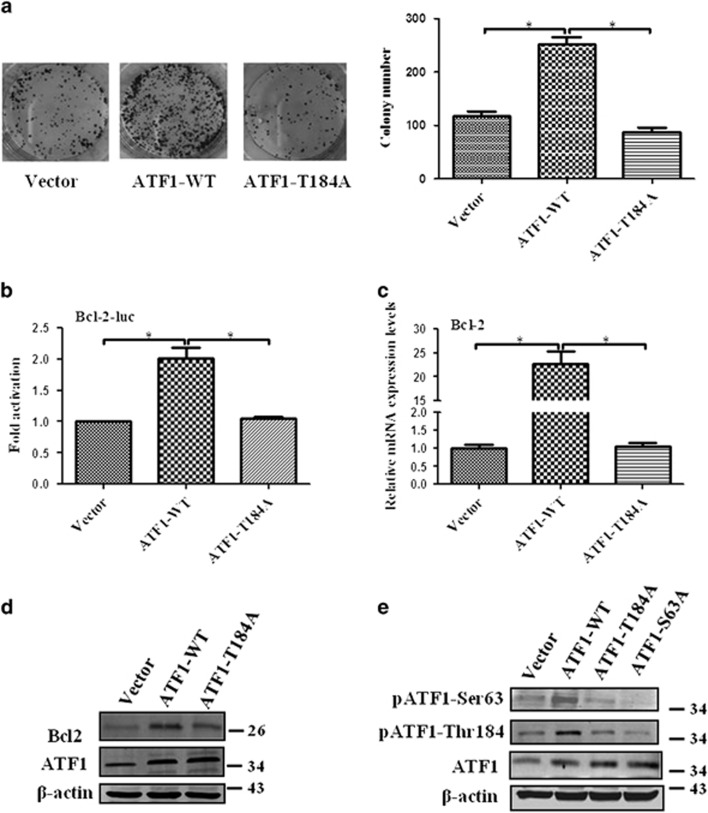
Mutation at Thr184 abrogates the biological function and transcriptional activity of ATF1. (**a**) Colony formation assay showed mutation at Thr184 abrogated the cell colony formation activity of ATF1 using CNE1 cells transfected with various plasmids. (**b-d**) Mutation at Thr184 abrogated the Bcl-2 transcriptional activity of ATF1 indicated by luciferase assay, qRT-PCR assay and western blotting using CNE1 cells transfected with various plasmids for 24 h. (**e**) Western blotting showed Ser63 and Thr184 affect their phosphorylations mutually using CNE1 cells transfected with various plasmids for 24 h. The remaining band was the endogenous phosphorylate proteins in the NPC cells as indicated in the first lane 'vector'. *P<0.05

As phosphorylated Thr184 was not reported previously, we investigated whether the biological function and transcriptional activity of ATF1 would be affected by Thr184 or not. The colony formation assay indicated that mutation at Thr184 impaired the cell colony formation ability of ATF1 (Figure 5a). The transcriptional activity of ATF1 was affected by mutation at Thr184. The relative luciferase activity of Bcl-2 reporter in Thr184 mutation cells was lower than that in ATF1 wild-type (WT) cells (Figure 5b). The mRNA and protein expression level of Bcl-2 in Thr184 mutation cells were lower than the level in ATF1 WT cells (Figures 5c and d). However, the confocal fluorescence analysis indicated that ATF1 was mainly expressed in the nucleus and the intranuclear accumulation of ATF1 was not affected by Thr184 mutation (Supplementary Figure S7). Interestingly, mutation at Thr184 impaired the phosphorylation of ATF1 at Ser63 and mutation at Ser63 also abolished the phosphorylation of ATF1 at Thr184 (Figure 5e).

### High expression of Pin1 in NPC tissue is positively correlated with ATF1

**Figure 6:**
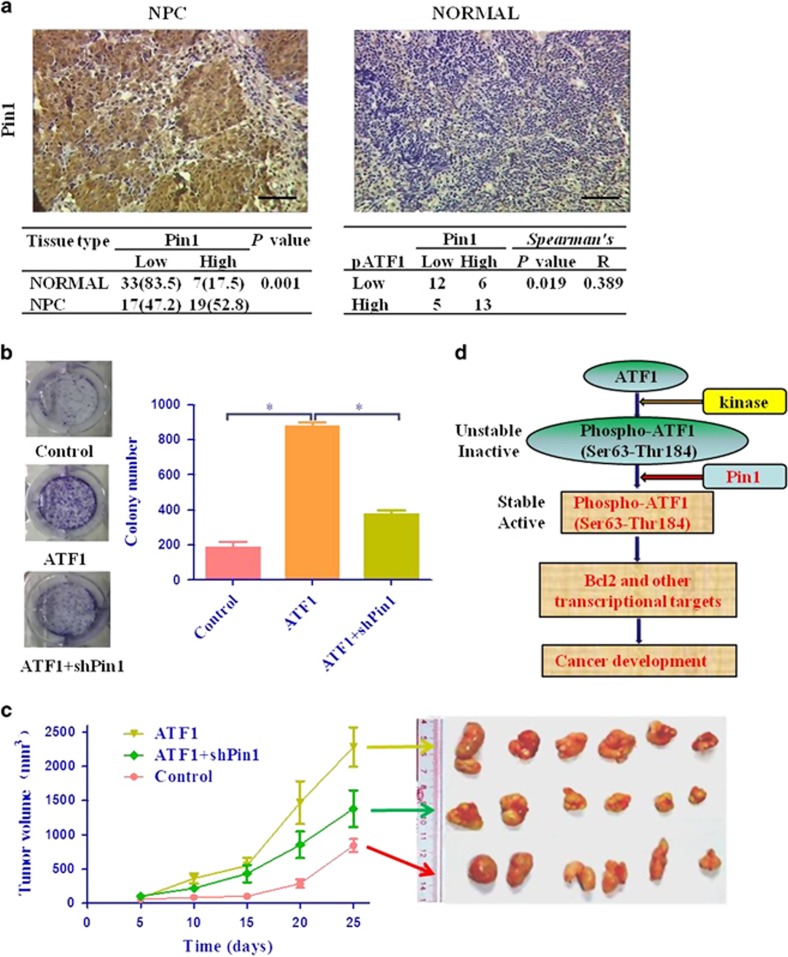
ATF1 function in NPC regulated by Pin1. (**a**) IHC staining of Pin1 in NPC tissues showed Pin1 expression was higher in NPC tissues and correlated with pATF1-Ser63. Scale bars, 50 *μ*m. (**b** and **c**) Overexpression of ATF1 promoted NPC tumorigenesis, which was attenuated by knockdown of Pin1 both *in vitro* and *in vivo*. (**b**) Colony formation assay showed co-expression with shPin1 impaired the cell colony formation ability of ATF1 using the stable cells established in CNE1. (**c**) The volumes of xenograft (six mice per group) were measured every 5 days after inoculation and tumors dissected at day 25 were imaged. (**d**) Schematic graph showed ATF1 function regulated by Pin1. *P<0.05

The expression of Pin1 in the NPC tissues and adjacent/normal specimens were examined using IHC staining. Pin1 expression was significantly higher in tumor tissues than adjacent/normal specimens (52.8% and 17.5%, respectively, *P*=0.001; Figure 6a). Furthermore, a significant association between the expression levels of Pin1 and phosphorylated ATF1 at Ser63 in NPC tissues was determined by Spearman's rank correlation test (Spearman's *R*=0.389, *P*=0.019; Figure 6a).

### ATF1 promoted NPC tumorigenesis is regulated by Pin1

To investigate whether knockdown of Pin1 affects the tumorigenesis promoted by ATF1, stable cells of CNE1 with ATF1 overexpression and combined with knockdown of Pin1 were established. The colony formation assay indicated that knockdown of Pin1 impaired the cell colony formation ability of ATF1 *in vitro* (Figure 6b). The results of xenograft experiments showed that overexpression of ATF1 in CNE1 cells (CNE1:ATF1) led to markedly increased tumor formation compared with the control CNE1 cells (Figure 6c). Importantly, simultaneous knockdown of Pin1 and overexpression of ATF1 in CNE1 cells (CNE1:ATF1/shPin1) led to tumor formation much slower than overexpression of ATF1 in CNE1 cells (CNE1:ATF1).

## Discussion

NPC is a highly invasive malignant tumor with high incidence rates in South-Eastern Asia and a number of provinces in South-Eastern China.^23^ Overexpression of ATF1 in NPC is found to be associated with clinical stage.^7^ However, the biological function of ATF1 in NPC is poorly investigated. Our data suggested a promotive role of ATF1 in NPC tumorigenesis both *in vitro* and *in vivo*. Although highly phosphorylated level of ATF1 was found in NPC, the GEO data indicated that the mRNA expression was not higher and mutation was rare. Therefore, we infer that the highly phosphorylated level of ATF1 is mediated by post-transcription regulation in NPC.

The mainly reported post-transcription regulatory mechanism of ATF1 is phosphorylation induced by serine/threonine kinases.^8^ It would be interesting to investigate whether the phosphorylation of ATF1 is modulated by the novel phosphorylation regulation by Pin1. In our study, a direct interaction between Pin1 and ATF1 was demonstrated by mammalian two-hybrid assay and coimmunoprecipitation experiments. On one hand, the expression of Pin1 substrate could be regulated by Pin1 in both the transcription level and protein level.^20^ Cyclin D1 is a paradigm that regulated by Pin1 in both the transcription level and protein level. Pin1 enhances the activation of the c-Jun/c-Fos, *β*-catenin and NF-*κ*b TFs, and thereby increases the transcription of cyclin D1.^16, 18, 19^ Pin1 can also stabilize the protein stability through directly binding to phosphorylated cyclin D1.^24^ On the other hand, Pin1 could regulate the protein level of its substrate without interfering the mRNA level. Pin1 binds to the pThr254-Pro motif in p65 resulting in increased nuclear accumulation and protein stability of p65.^18^ Our data further suggested that Pin1 enhanced protein stability of ATF1 by CHX treatment.

Our results indicated that ATF1 promoted NPC tumorigenesis is regulated by Pin1. However, ATF1 is not the only protein regulated by Pin1. As shown in Supplementary Figure S3, overexpression of Pin1 increased the colony formation ability in ATF1 knockdown cells, even compared with the control. A major reason for this observation is that Pin1 modulates other phosphorylated proteins, such as p53, p65, cyclin D1, c-Jun, c-Fos, NF-*κ*B and *β*-catenin,^14, 15, 16, 17, 18, 19^ which overcome the effect by knockdown of ATF1.

Bcl-2 is an important regulator of cell death.^25^ Bcl-2 promoter contains binding sites for CREB/ATF1. The phosphorylation of CREB and ATF1 is reported to be required for the upregulation of Bcl-2.^2^ In this study, our data indicated that Pin1 could upregulate Bcl-2 transcription through ATF1. Interestingly, the Bcl-2 luciferase reporter activity was not statistically significant different between Pin1-overexpressing cells and control, whereas the mRNA and protein expression level of Bcl-2 in Pin1-overexpressing cells was higher. This was mainly because the Bcl-2 luciferase reporter contained a short promoter region that only regulated by ATF1. The luciferase activity could not be regulated by Pin1 directly. However, Bcl-2 is also a direct target of Pin1,^26^ and Pin1 could regulate the endogenous level of Bcl-2.

To activate its transcriptional activity, ATF1 is mainly phosphorylated at Ser63.^27^ Phosphorylation of ATF1 at Ser63 enforce its binding to the ATF/CRE site.^9^ However, a recent study indicates that ATF1 could be phosphorylated at Ser198 but not Ser63 by HIPK2 (homeodomain-interacting protein kinase 2), a DNA-damage-responsive nuclear kinase, leading to transcriptional repression of ferritin H.^8^ Our results suggested the ATF1 Thr184 would be a novel phosphorylated site, which was important for the interaction between ATF1 and Pin1. Although the phosphorylated Ser63 is not a direct binding site with Pin1, Ser63 could modulate the protein tertiary structure of ATF1 and therefore (1) affect the phosphorylation of Thr184; (2) affect the interaction between Pin1 and ATF1 at Thr184. Our data further indicated that mutation at Thr184 impaired the biological function and transcriptional activity of ATF1. MAPK signaling is the chief kinase in activating ATF1 function.^10^ However, whether the phosphorylation of ATF1 at Thr184 could be induced by these kinases is unclear. It is important to identify the kinase stimulating phosphorylation at Thr184 in the future.

As our results indicated that Pin1 was a novel regulator of ATF1, we next investigated the effect of Pin1 on the biological function of ATF1 in NPC. Higher expression of Pin1 was found to be correlated with ATF1 in NPC tissue. Colony formation assay and xenograft experiment indicated that the ATF1 promotion of NPC tumorigenesis was regulated by Pin1 both *in vitro* and *in vivo*. Overall, our study suggests that Pin1 is a novel regulator of ATF1 at Thr184 and thereby enhances ATF1 transcription activity and tumorigenesis promotive function in NPC.

## Materials and Methods

### Antibodies and reagents

Antibodies against Pin1, ATF1 were purchased from Santa Cruz Biotechnology, Inc (Santa Cruz, CA, USA); against phosphorylated ATF1 (Ser63) was from Abcam, Inc (Cambridge, MA, USA). Infrared-dye-conjugated secondary antibodies were from Rockland Immunochemicals (Gilbertsville, PA, USA). JetPEI transfection reagent was from Polyplus (llkirch, France). A polyclonal antibody against phosphorylated ATF1 (Thr184) was raised in a rabbit. Specificity of the antibody was confirmed by dot blotting and ELISA assay (Supplementary Information,Supplementary Figure S4).

### Cell culture

The NPC CNE1 and CNE2 cell lines derived from well differentiated and undifferentiated NPCs from Chinese patients, respectively, and HEK-293 cell were either obtained from the nitrogen stock in our department or purchased from the Cell Bank (Institute of Life Science, Chinese Academy of Science, Shanghai, China). Pin1^+/+^ and Pin1^−/−^ mouse embryonic fibroblast (MEF) cells were kindly provided by Professor Dong (The Hormel Institute, University of Minnesota, Austin, MN, USA) and originally generated by Fujimori *et al.*^28^ The CNE1 and CNE2 were cultured in RPMI 1640 medium (Gibco, Inc, Shanghai, China); Pin1^+/+^, Pin1^−/−^ and HEK-293 cell cultured in DMEM medium (Gibco) supplemented with 10% fetal bovine serum (FBS) at 37 °C in a humidified chamber containing 5% CO_2_. The immortalized nasopharyngeal epithelial cell line NP69 (kindly provided by Professor Zeng from Sun Yat-Sen University Cancer Center, Guangdong, China) was cultured in keratinocyte serum-free medium (Invitrogen, Carlsbad, CA, USA) supplemented with 5% FBS and 0.2 ng/ml recombinant epidermal growth factor.

### Plasmid constructs, transfection, lentiviral infection and stable cells

Pin1-ORF and ATF1-ORF were polymerase chain reaction (PCR)-amplified and cloned into expression vector pcDNA6.0/myc-His B vector (Invitrogen) to produce the myc and His epitope-tagged construct. The vector of ATF1 mutants Ser63Ala, Thr184Ala, Ser198Ala, Ser63Ala-Thr184Ala, Ser63Ala-Ser198Ala, Thr184Ala-Ser198Ala and Ser63Ala-Thr184Ala-Ser198Ala were generated using the KOD-Plus-Mutagenesis kit (Toyobo Co., Ltd, Osaka, Japan). To construct the shRNA-Pin1 (shPin1) or shRNA-ATF1 (shATF1), the mU6pro vector was digested with *Xba*I and *Bbs*I. The mU6pro vector contained the mouse U6 snRNA promoter (RNA polymerase III) with a Bbs1-cloning site, which allowed insertion of siRNA (shRNA) template sequences.^29^ The annealed synthetic primers (Pin1 shRNA sense: 5′- TTTG*GCTCAGGCCGAGTGTACTA*TTCAAGAGA*TAGTACACTCGGCCTGAGC*TTTTT-3′ and antisense: 5′-CTAGAAAAA*GCTCAGGCCGAGTGTACTA*TCTCTTGAA*TAGTACACTCGGCCTGAGC*-3′ ATF1 shRNA sense: 5′-TTTG*GATCCGAACTACACCTTCA*TTCAAGAGA*TGAAGGTGTAGTTCGGATC*TTTTT-3′ and antisense: 5′-CTAGAAAAA*GATCCGAACTACACCTTCA*TCTCTTGAA*TGAAGGTGTAGTTCGGATC*-3′. Letters in italics and underlines implicated the target sequences.) were then introduced into the mU6pro vector. The target sequences for Pin1 and ATF1 were from two studies.^30, 31^ Another siRNAs for Pin1 and ATF1 (from Santa Cruz Biotechnology) were used to avoid off-target of shRNA (Supplementary Figure S5). DNA fragments of 5′-flanking region of the human Bcl-2 gene (–1640 to –1526)^32^ were synthesized and inserted into a pGL3 luciferase reporter vector (Promega, Inc, Beijing, China). The recombinant plasmids were confirmed by agarose gel electrophoresis and DNA sequencing.

The plasmid was transfected into NPC CNE1 or CNE2 cell using jetPEI (polyplus-transfection) according to the manufacturer's instructions. To generate CNE1:ATF1 and CNE1:ATF1/shPin1 cells, CNE1 cells were infected with ATF1 and shPin1 lentiviral particles (purchased from GenePharma Co., Ltd, Shanghai, China. The lentivirus packaging vectors were LV5 and pGLV3 plasmid, respectively. More information could be found in the website of GenePharma Co., Ltd) sequentially, and screened with 1 *μ*g/ml puromycin. To generate NP69:ATF1, NP69:Pin1 and NP69:ATF1/Pin1 cells, NP69 cells were infected with ATF1 and Pin1 lentiviral particles, respectively, or sequentially, and screened with 1 *μ*g/ml puromycin. Western blot was used to verify transfection (Supplementary Figure S6).

### Xenograft experiments

BALB/C nude mice, at an age of 6–8 weeks at the time of inoculation, were used to establish xenografts. The sample size was 18. Mice were randomized into three groups (*n*=6 per group) and there were no exclusion criteria. All data were analyzed unblinded and verified by two independent researchers. Cells (2 × 10^6^/0.1 ml) were inoculated subcutaneously into the left flank of each nude mice. The volumes of xenografts were measured every 5 days after inoculation. The tumor volumes were calculated according to the formula: volume=D × d^2^/2, where D and d are the longest and the shortest diameters, respectively. The xenografts were dissected and weights were measured 25 days after inoculation. Animal experiments were approved by Animal Care and Use Committee of Shenzhen Third People's Hospital.

### Cell counting kit-8 (CCK-8) assay

Cell growth rates were detected by CCK-8 (Dojindo Laboratories, Kumamoto, Japan) assay as previously described.^33^ CNE1 stable Pin1-expressing clones or CNE2 cells transfected with shPin1 plasmids were seeded in 96-well plates (1 × 10^3^ per well). NP69 cells were seeded in 96-well plates (4 × 10^3^ per well). After culturing for different periods of time, CCK-8 solution (10 *μ*l per 100 *μ*l medium) was added to each well, and cells were then incubated for 1 h at 37 °C. Absorbance was measured at 450 nm using Synergy^2^ Multi-Mode Microplate Reader (BioTek, Winooski, VT, USA). Five replicate wells for each sample and three parallel experiments were performed.

### Colony formation assay

The colony formation potential of the introduced genes in cells was evaluated by colony formation assay as previously described.^33^ CNE1 stable Pin1-expressing clones or CNE2 cells transfected with shPin1 plasmids were seeded in six-well plates (500 or 1 × 10^3^ per well). NP69 cells were seeded in six-well plates (500 per well). After culturing for 2 weeks, surviving colonies (>50 cells per colony) were counted with crystal violet staining. Triplicate independent experiments were performed.

### Protein extraction, western blotting and coimmunoprecipitations

Total protein was extracted with RIPA lysis buffer (Beyotime Ins. Bio, Shanghai, China). Protein concentration was determined by the bicinchoninic acid assay (Pierce, Inc, Rockford, IL, USA). Equal amount of whole-cell lysates were resolved by SDS-PAGE and transferred to a polyvinylidene difluoride membrane (Millipore, Billerica, MA, USA). The membrane was probed with the primary antibodies overnight at 4 °C and incubated with infrared-dye-conjugated secondary antibodies for 1 h at room temperature. Protein bands were visualized by Odyssey Infrared Imaging System (LI-COR Biotechnology, Lincoln, NE, USA). To investigate the interaction between Pin1 and ATF1 at the endogenous level, the clarified lysates were first incubated with anti-Pin1 or anti-ATF1 for 2 h at 4 °C. Then, protein A/G-agaroses were added overnight, and precipitates were washed four times with lysis buffer and analyzed by western blotting.

For protein stability assays, exponentially growing cells were treated with 50 *μ*g/ml CHX (Sigma, Inc, Shanghai, China) and harvested at various hours post-CHX treatment. Cell protein was extracted as described above, followed by immunoblotting.

### Mammalian two-hybrid assay

The assay was performed as previously described.^34^ Cells were seeded into 48-well plates and incubated with 10% FBS-DMEM for 18 h before transfection. The DNAs, pACT-ATF1, pBIND-Pin1 and pG5-luciferase were combined in the same molar ratio and the total amount of DNA was not >100 ng per well. The transfection was done using jetPEI following the manufacturer's recommended protocols. The cells were lysed and assayed for luciferase activity using the Dual Luciferase kit (Promega) according to the manufacturer's instructions. The relative luciferase activity was calculated and normalized based on the pG5-luciferase basal control.

### ELISA assay

A modified ELISA assay was performed to demonstrate the specific binding of Pin1 to phosphorylated T184 peptides *in vitro*. In the modified ELISA assay, the synthesized peptides (pT184 or T184) were diluted and coated on the wells of a PVC microtiter plate at 4°C overnight. After washing three times with PBS, the plate was blocked with 10% normal bovine serum in PBS. The Pin1 recombinant protein (Abcam) was added to the plate before the antibody for Pin1 was added. The plate was incubated with HRP-conjugated secondary antibody at 37 °C for 30 min and visualized with 3,3',5,5'-tetramethylbenzidine. Absorbance was measured at 450 nm using Synergy^2^ Multi-Mode Microplate Reader (BioTek).

### Reporter gene assay

The luciferase reporter gene assay was performed as previously described.^33^ Cells were co-transfected with Bcl-2 reporter gene and Pin1, ATF1 expression plasmids. The pRL-TK vector-expressing (Promega) Renilla luciferase was co-transfected to calibrate the firefly luciferase activity. Cells were lysed and assayed for luciferase activity using the Dual Luciferase kit (Promega) according to the manufacturer's instructions. Triplicate experiments were done.

### RNA isolation and quantitative RT-PCR

These procedures were performed as described previously.^35^ Briefly, cells transfected with Pin1 or ATF1 expression plasmids were collected and RNAs were extracted using TRIzol (Invitrogen) according to the manufacturer's instructions. The primers used for amplifying Pin1, ATF1, Bcl-2 and the house keeping gene, glyceraldehyde-3-phosphate dehydrogenase (GAPDH) were listed as follow: Pin1-F, 5′-GAGCTGATCAACGGCTACATCC-3′ Pin1-R, 5′-AATGGCTTCTGCATCTGACCTCT-3′ ATF1-F, 5′-CAACTATTCTTCAGTATGCACAGACC-3′ ATF1-R, 5′-GTTTGCATATCTCCTGATGCAGTT-3′ Bcl-2-F, 5′-GAGGATTGTGGCCTTCTTTG-3′ Bcl-2-R, 5′-GCCGGTTCAGGTACTCAGTC-3′ GAPDH-F, 5′-CTCCTCCTGTTCGACAGTCAGC-3′ GAPDH-R, 5′-CCCAATACGACCAAATCCGTT-3′.

### TMA and IHC analysis

NPC tissue microarrays (TMAs), containing 36 cases (most cases were in the advanced tumor stage, 35/36; 31 cases with matched normal tissues) of primary tumors, 17 cases of lymph node metastatic tumors and 12 cases of normal or reactive nasopharyngeal mucosal tissues, were purchased from US Biomax (Rockville, MD, USA: catalog no. NPC961). Informed consent from each subject and approval from the Institutional Ethics Committee of Guangdong Medical College were obtained. The IHC analysis was performed as previously described.^36^ The TMA slides were deparaffinized in xylene and rehydrated in a graded ethanol series. For antigen retrieval, the slides were boiled in EDTA (1 mmol/l; pH8.0) for 15 min in a microwave oven. Endogenous peroxidase was quenched with 3% hydrogen peroxide and nonspecific antigen was blocked with 10% normal goat serum. The sections were incubated with the primary antibodies against Pin1 or phospho-ATF1 (Ser63) overnight at 4°C. The primary antibodies were replaced by PBS as negative controls. The slides were incubated with HRP-conjugated secondary antibody (ChemMate for 30 minat room temperature and visualized with 3,3'-diaminobenzidine tetrahydrochloride) according to the manufacturer's instruction. The specimens were counterstained with hematoxylin.

The sample evaluation and information recording were conducted in a double-blinded manner. Two pathologists without knowing patients' information were responsible for assessing the results. The staining score was based on its intensity (0, no staining; 1, weak staining; 2, moderate staining; 3, strong staining) and the percentage of positive cells (0, <5% positive cells; 1, 5–25% positive cells; 2, 25–50% positive cells; 3,>50% positive cells). The final expression score was calculated from ‘intensity' multiplied by ‘percentage': ‘–' stands for scores 0–1, ‘+' for scores 2–3, ‘++' for scores 4–6 and ‘+++' for scores >6. The cases scored as ‘–' and ‘+' were combined as low score and the cases with scored as ‘++' and ‘+++' were combined as high score for statistical analysis.

### Mutation analysis of ATF1 and mRNA expression in NPC

We used GEO data repository Sequence Read Archive (SRP035573) to analyze the mutation status of ATF1 in NPC.^37, 38^ This archive included whole-exome sequencing data of 56 NPC germline and tumor pairs. Blast analysis was performed to compare the ATF1 sequence with the sequencing data of these samples. Another GEO data repository (GSE 13597) was used to analyze the mRNA expression of ATF1 in NPC.^37, 39^ This archive included a genome-wide mRNA expression data of 25 NPC and 3 non-tumor tissues.

### Statistical analysis

Quantitative value was represented as mean±S.D. Student's *t*-test was used to compare the mean value of each group. The relationship between Pin1 and ATF1 phosphorylation expression, as well as clinicopathological characteristics was analyzed using Chi-square test or Fisher's exact tests. The SPSS software package (SPSS, Inc. Chicago, IL, USA) and GraphPad Prism (GraphPad Software, Inc. La Jolla, CA, USA) were used for the statistical analysis and data plotting. *P*<0.05 was considered as statistically significant.
