# Supplementary material for: The protein level and transcription activity of activating transcription factor 1 is regulated by prolyl isomerase Pin1 in nasopharyngeal carcinoma progression
Source: Cell Death Dis. 2016 Dec 29;7(12):e2571–. doi: 10.1038/cddis.2016.349 (PMC5260992; doi:10.1038/cddis.2016.349)
Supplement: Supplementary Information [file cddis2016349x1.doc]

Supplementary Information includes 1.) a doc file describing the supplementary Materials and Methods, Results, Figure legends and Tables; 2.) seven figure files. 3.) a pdf file with cell authentication reports.

**Materials and Methods**

**Antibodies and reagents**

A peptide including phosphorylated ATF1 (Thr184) MQTYQIRT(p)TPSATSLPC was synthesized and used to immunize a rabbit (NZB white, female) by four subcutaneous injections, 2 week apart. The resulting serum was subjected to affinity-purification. This antibody did not crossreact with the non-phosphorylated counterpart of ATF1.

**Antibody validation by ELISA and dot blot assay**

In ELISA assay, the synthesized antigen (pT184 or T184) was diluted and coated on the wells of a PVC microtiter plate at 4℃ overnight. After washing three times with PBS, the plate was blocked with 10% normal bovine serum in PBS. The purified antibody was added in a series of dilutions and incubated at 37℃ for 30 min. The plate was incubated with HRP-conjugated secondary antibody at 37℃ for 30 min and visualized with 3,3',5,5'-Tetramethylbenzidine (TMB). Absorbance was measured at 450 nm using Synergy^2^ Multi-Mode Microplate Reader (BioTek, Winooski, VT, USA).

In dot blot assay, 5 μl synthesized antigen (pT184 or T184) in 1 mg/ml PBS was spot onto the PVDF membrane respectively. Non-specific sites were blocked by 5% non-fat milk in TBST at room temperature for 1 hour. The membrane was incubated with the purified antibody (1:4000) at room temperature for 1 hour and infrared-dye-conjugated secondary antibodies for 1 hour at room temperature. The blot images were visualized by Odyssey Infrared Imaging System (LI-COR Biotechnology, Lincoln, NE, USA).

**Confocal Laser-scanning Fluorescence Microscopy**

The confocal laser-scanning fluorescence analysis was used to examine the intranuclear accumulation of ATF1 in CNE1 cells transfected with wild-type ATF1 and ATF1-T184A upon MG-132 treatment. The NPC cells CNE1 were transfected with wild-type ATF1 or ATF1-T184A for 36 hour and then treated with MG-132 for 0 hour, 4 hour and 8 hour. The Cells were fixed in 4% formalin for 15 min and blocked for 15 min in 1% donkey serum in 0.3% Tween in PBS. The CNE1 cells were incubated 1 h with anti-ATF1 rabbit antibody. After washing with PBS, cells were incubated with secondary antibody (Dylight 550 goat anti-rabbit IgG) and Hoechst. Fluorescence was observed by laser-scanning confocal microscopy (Leica, Solms, Germany).

**Results**

**Specificity of the antibody was confirmed with ELISA and dot blot assay**

The ELISA assay showed extremely low binding ability of the purified antibody with the non-phosphorylated antigen T184 (Figure S4A). The binding ability of the purified antibody with the phosphorylated antigen pT184 was much higher than that with the non phosphorylated antigen T184. The binding ability decreased along with the dilutions. The dot blot assay showed high binding ability of the purified antibody with the phosphorylated antigen pT184 whereas little binding with the non-phosphorylated antigen T184 (Figure S4B). These results indicated that the purified antibody recognized the phosphorylated antigen pT184 specifically.

**ATF1 promoted NPC tumorigenesis is regulated by Pin1**

Co-expression of Pin1 and ATF1 in NP69 cells (an immortalized nasopharyngeal epithelial cell line) strengthened the proliferation effect and colony formation ability of ATF1 (Figure S2). Over-expression of Pin1 rescued the colony formation impairment of shATF1 in NPC cells (Figure S3). These data suggest that ATF1 is critical for tumor formation in NPC and Pin1 modulates the tumorigenesis promoted by ATF1.

**Figure Legends,**

**Figure S1.** ATF1 transcriptional activation of Bcl-2 is impaired by knockdown of Pin1 in the liver cancer cell line HepG2 and lung cancer cell line A549 transfected with various plasmids for 48 hours.

**Figure S2.** Co-expression of Pin1 enhances the proliferation effect and colony formation ability of ATF1 in NP69 cells, an immortalized nasopharyngeal epithelial cell line. Stable cells with various modifications established in NP69 were used. (**A**) CCK-8 assay showed the cell growth of NP69 with various modifications. (**B**) colony formation assay showed the cell colony formation ability of NP69 with various modifications. *, *P* < 0.05.

**Figure S3.** Colony formation assay showed Pin1 rescued the cell colony formation ability impaired by ATF1 knock-down using CNE1 cells transfected with various plasmids for 48 hours. *, *P* < 0.05.

**Figure S4.** Specificity of the antibody against phosphorylated ATF1 (Thr184) is confirmed by dot blotting and ELISA assay. (**A**) The titer and specificity of the purified antibody was determined by ELISA. The coating antigens were MQTYQIRTT(p)PSATSLP (pT184) or MQTYQIRTTPSATSLP (T184) respectively. (**B**) The specificity of the purified antibody determined by dot blot. The spotted antigens were MQTYQIRTT(p)PSATSLP (pT184) or MQTYQIRTTPSATSLP (T184) respectively.

**Figure S5.** Another siRNAs for Pin1 and ATF1 (from Santa Cruz Biotechnology) were used to avoid off-target of shRNA. (A) Colony formation assay showed the cell colony formation ability of CNE2 cells with ATF1 knock-down. (B) Pin1 knockdown cells and control CNE2 cells were treated with 50 μg/ml cycloheximide (CHX) for indicated durations followed by WB analysis. The quantitative data of ATF1 protein are represented in the right panel. (**C**) Luciferase assays showed Bcl-2 promoter activity regulated by various modifications of ATF1 and Pin1. (**D**) qRT-PCR assays showed Bcl-2 mRNA regulated by various modifications of ATF1 and Pin1. (**E**) Western blotting assays showed Bcl-2 protein regulated by various modifications of ATF1 and Pin1.

**Figure S6.** Western Blot verification of transfection. (A) Verification of transfection in Fig 1B. (B) Verification of transfection in Fig 1C. (C) Verification of transfection in Fig 1D. (D) The expression of Pin1 in Pin1 +/+ and Pin1 -/- as shown in Fig 3D. (E) Verification of transfection in Fig 6B. (F) Verification of transfection in Fig S2. (G) Verification of transfection in Fig S3.

**Figure S7.** The intranuclear accumulation of ATF1 in CNE1 cells transfected with wild-type ATF1 (ATF1-WT) and ATF1-T184A upon MG-132 treatment using confocal microscopy detection. Scale bars, 10 μm.

**Supplementary Tables**

| Table S1. Summary of variations of ATF1 in 56 pairs of NPCs using GEO data. | | | | | | | | | |
| --- | --- | --- | --- | --- | --- | --- | --- | --- | --- |
| Patient ID | Position  in ATF1 | Allele  in ATF1 | Germline | | |  | Tumor allele | | |
|  |  |  | Allele | No.of  reads | No.with variation |  | Allele | No.of  reads | No.with variation |
|  |  |  |  |  |  |  |  |  |  |
| NPC2F | 320 | C | N.A. |  |  |  | A | 20 | 4 |
| NPC2F | 468 | G | N.A. |  |  |  | T | 23 | 1 |
| NPC2F | 479 | C | N.A. |  |  |  | A | 23 | 1 |
| NPC2F | 445 | A | N.A. |  |  |  | G | 23 | 1 |
| NPC2F | 601 | C | N.A. |  |  |  | A | 20 | 1 |
| NPC2F | 598 | T | N.A. |  |  |  | G | 20 | 1 |
| NPC2F | 718 | C | N.A. |  |  |  | A | 21 | 1 |
| NPC2F | 663 | C | N.A. |  |  |  | T | 10 | 1 |
| NPC23F | 217 | T | G | 1 | 1 |  | N.A. |  |  |
| NPC23F | 311 | G | T | 13 | 1 |  | N.A. |  |  |
| NPC23F | 743 | T | G | 49 | 1 |  | N.A. |  |  |
| NPC23F | 860 | A | T | 14 | 1 |  | N.A. |  |  |
| NPC23F | 354 | C | A | 13 | 1 |  | N.A. |  |  |
| NPC88D | 387 | C | C | 18 | 0 |  | A | 23 | 2 |
| NPC88D | 732 | A | A | 50 | 0 |  | T | 36 | 1 |
| NPC88D | 728 | C | C | 13 | 0 |  | A | 13 | 1 |
| NPC88D | 728 | C | C | 18 | 0 |  | T | 13 | 1 |
