# Supplementary figures and images for: The protein level and transcription activity of activating transcription factor 1 is regulated by prolyl isomerase Pin1 in nasopharyngeal carcinoma progression
Source: Cell Death Dis. 2016 Dec 29;7(12):e2571–. doi: 10.1038/cddis.2016.349 (PMC5260992; doi:10.1038/cddis.2016.349)

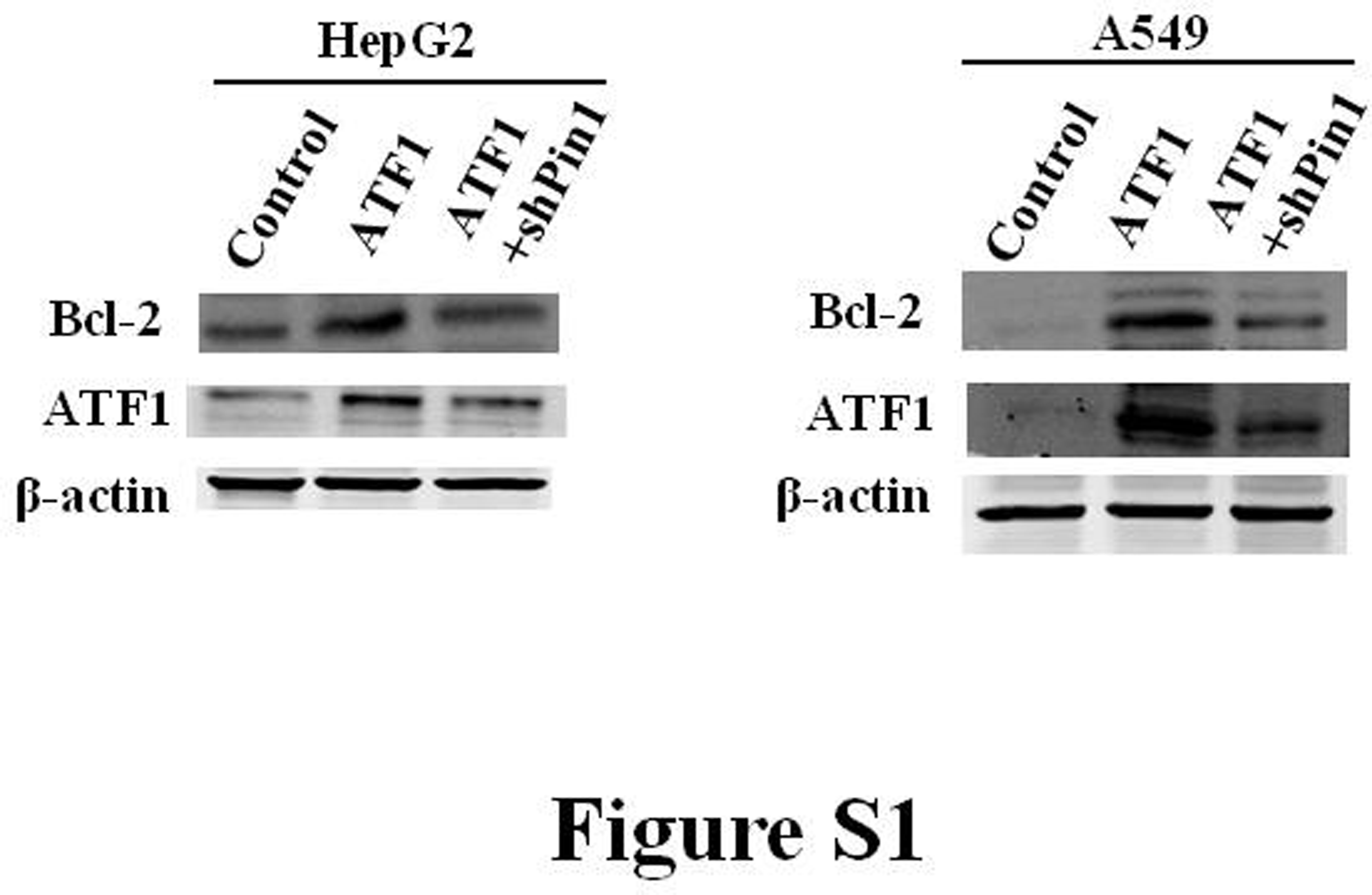

Supplement: Supplementary Figure 1 [file cddis2016349x2.tif]

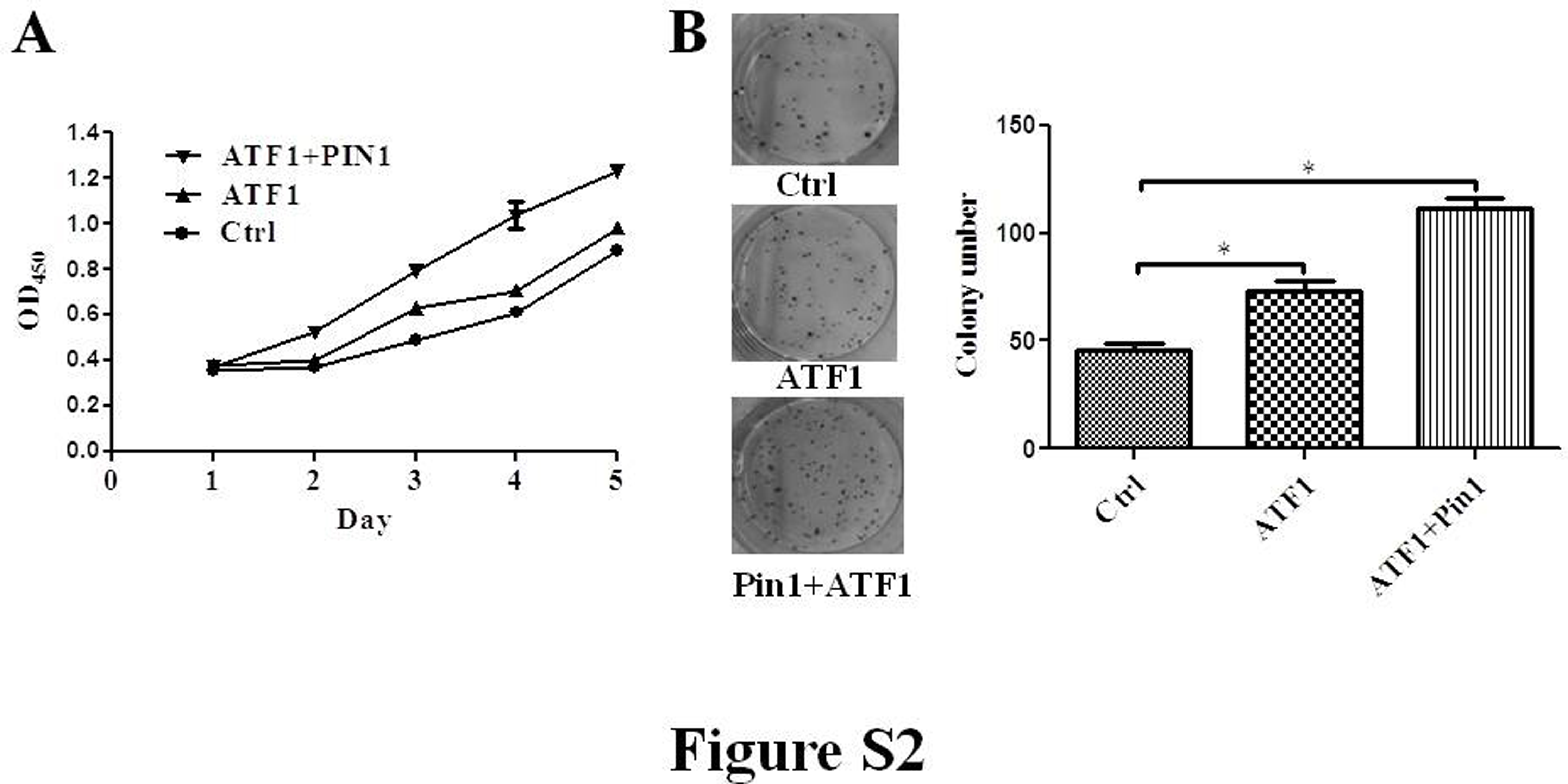

Supplement: Supplementary Figure 2 [file cddis2016349x3.tif]

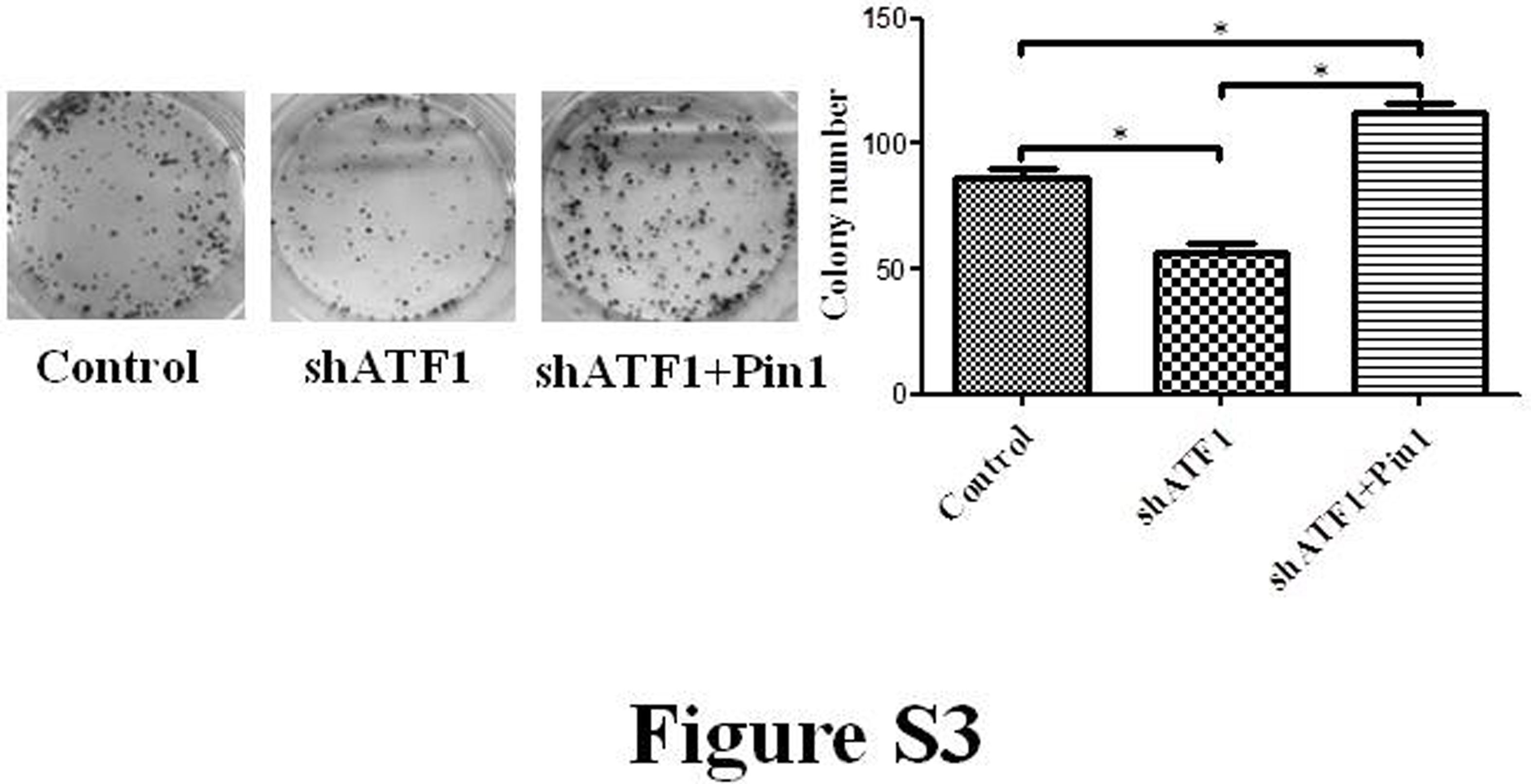

Supplement: Supplementary Figure 3 [file cddis2016349x4.tif]

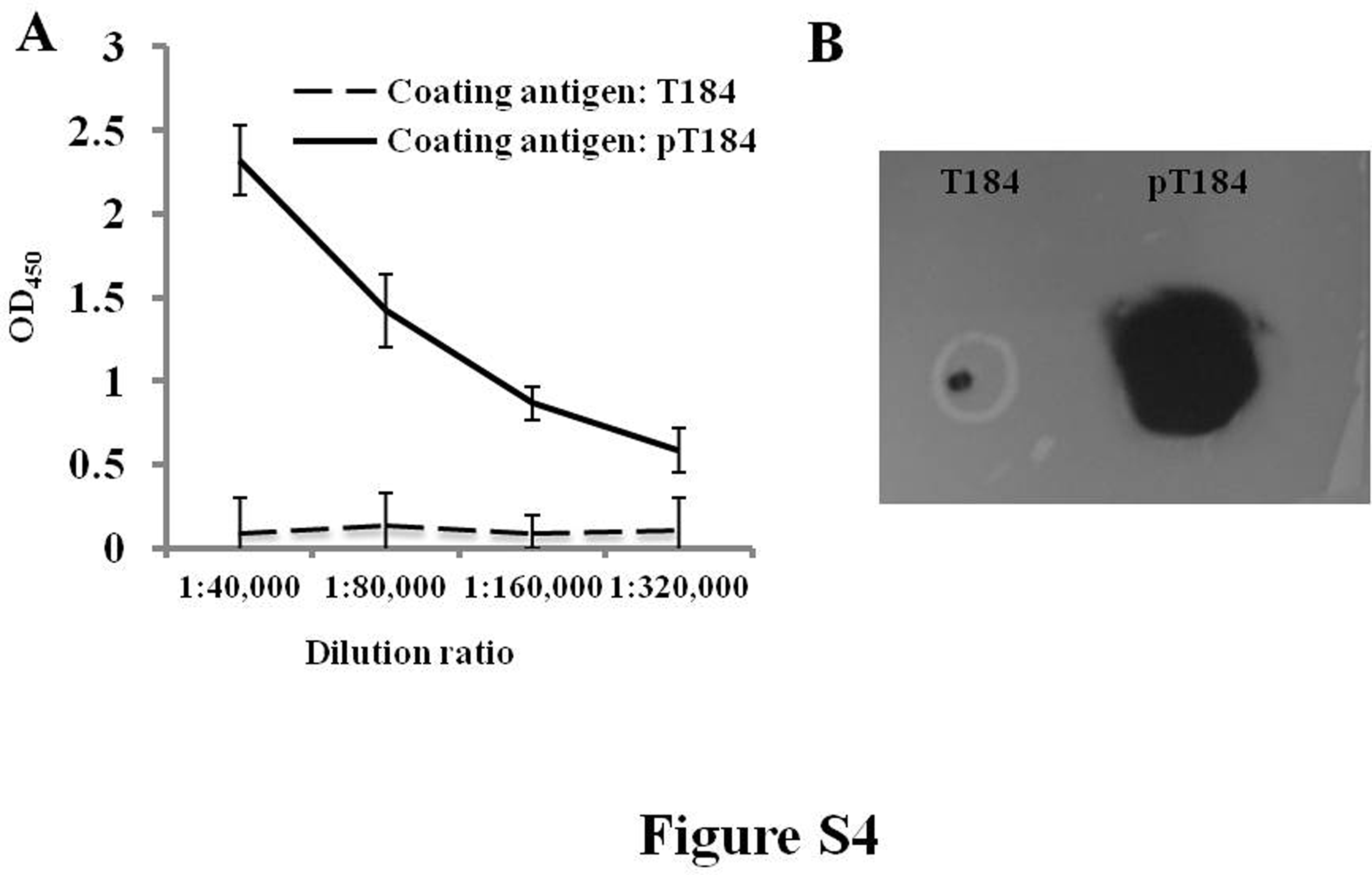

Supplement: Supplementary Figure 4 [file cddis2016349x5.tif]

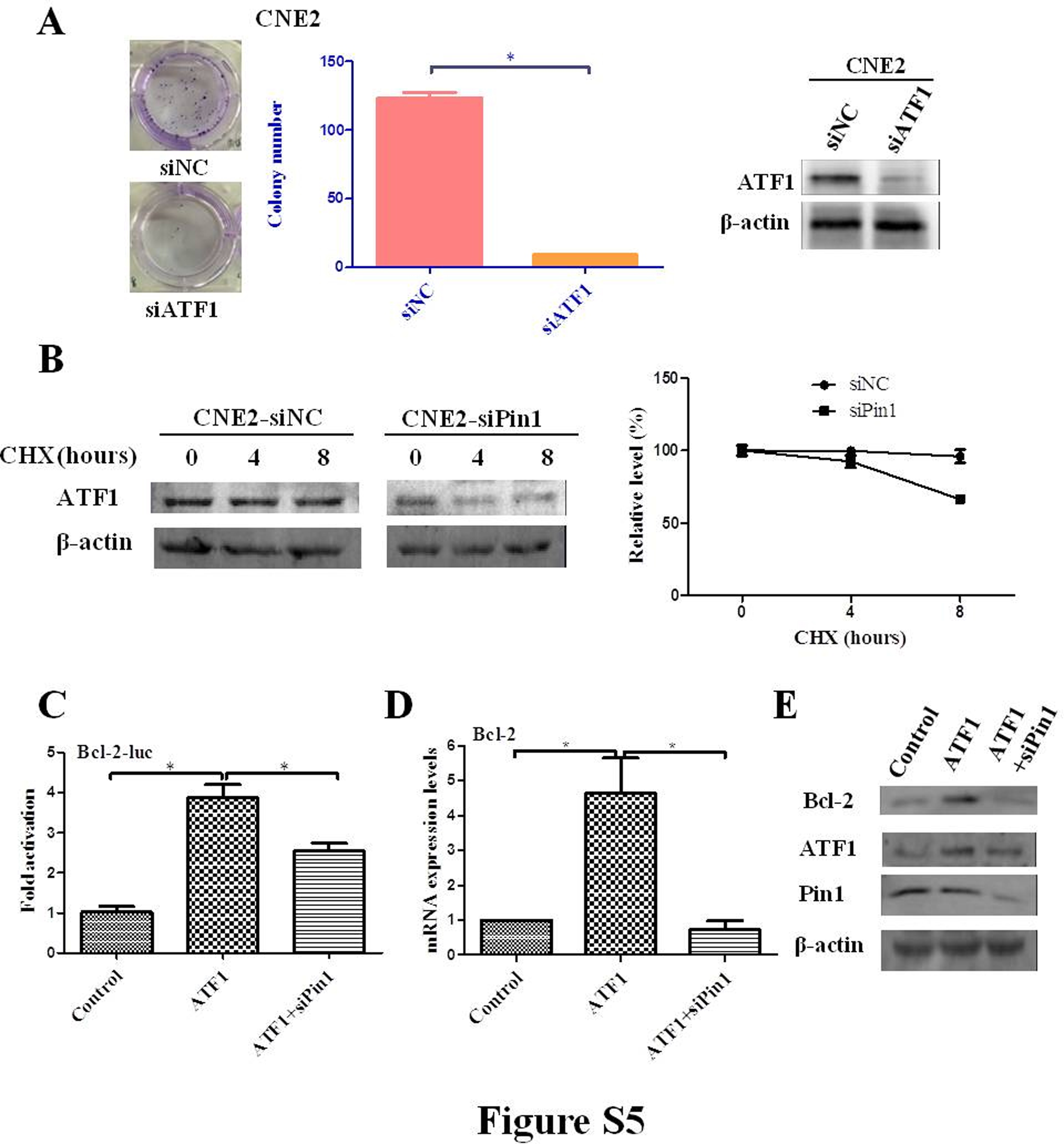

Supplement: Supplementary Figure 5 [file cddis2016349x6.tif]

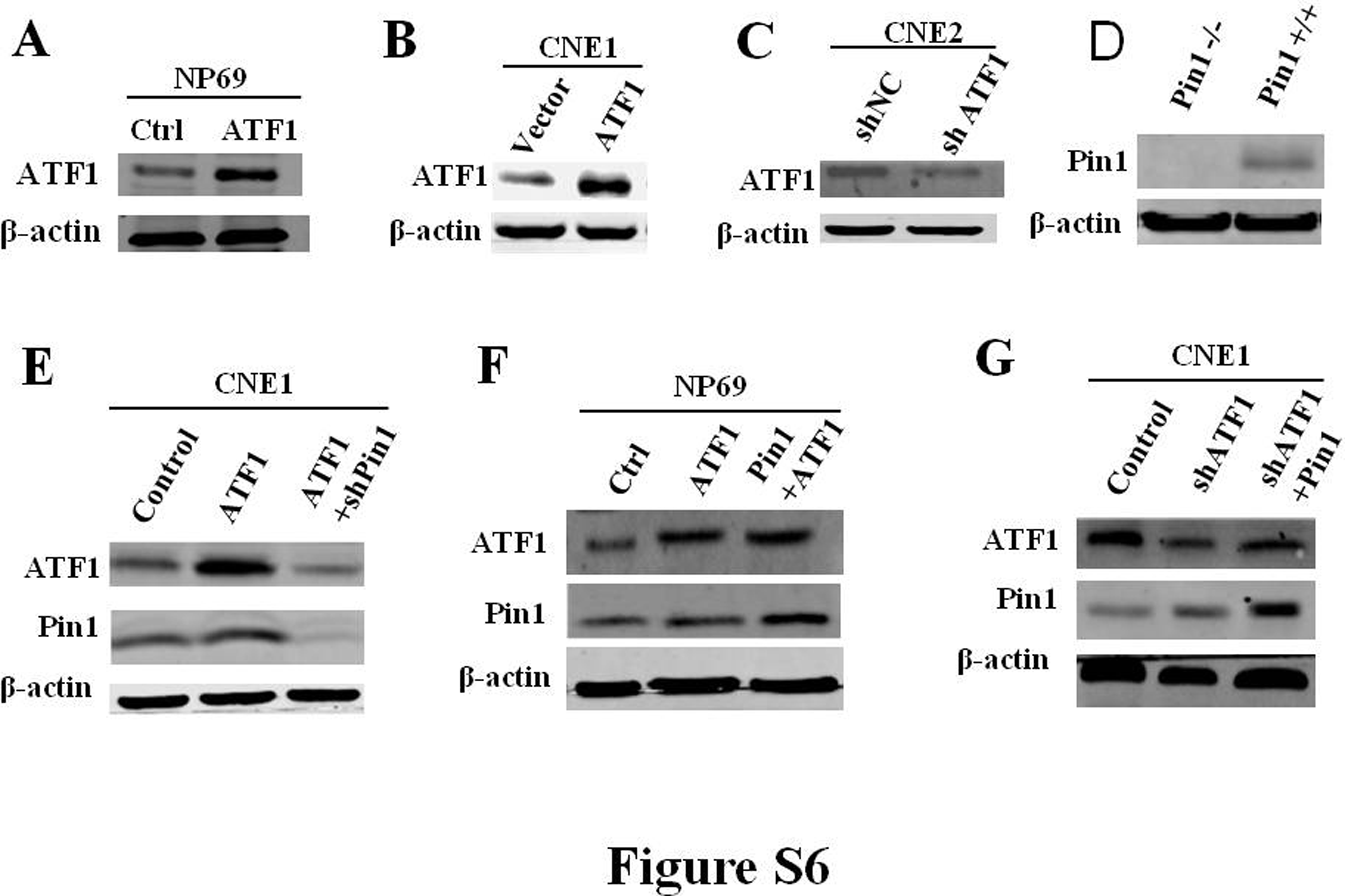

Supplement: Supplementary Figure 6 [file cddis2016349x7.tif]

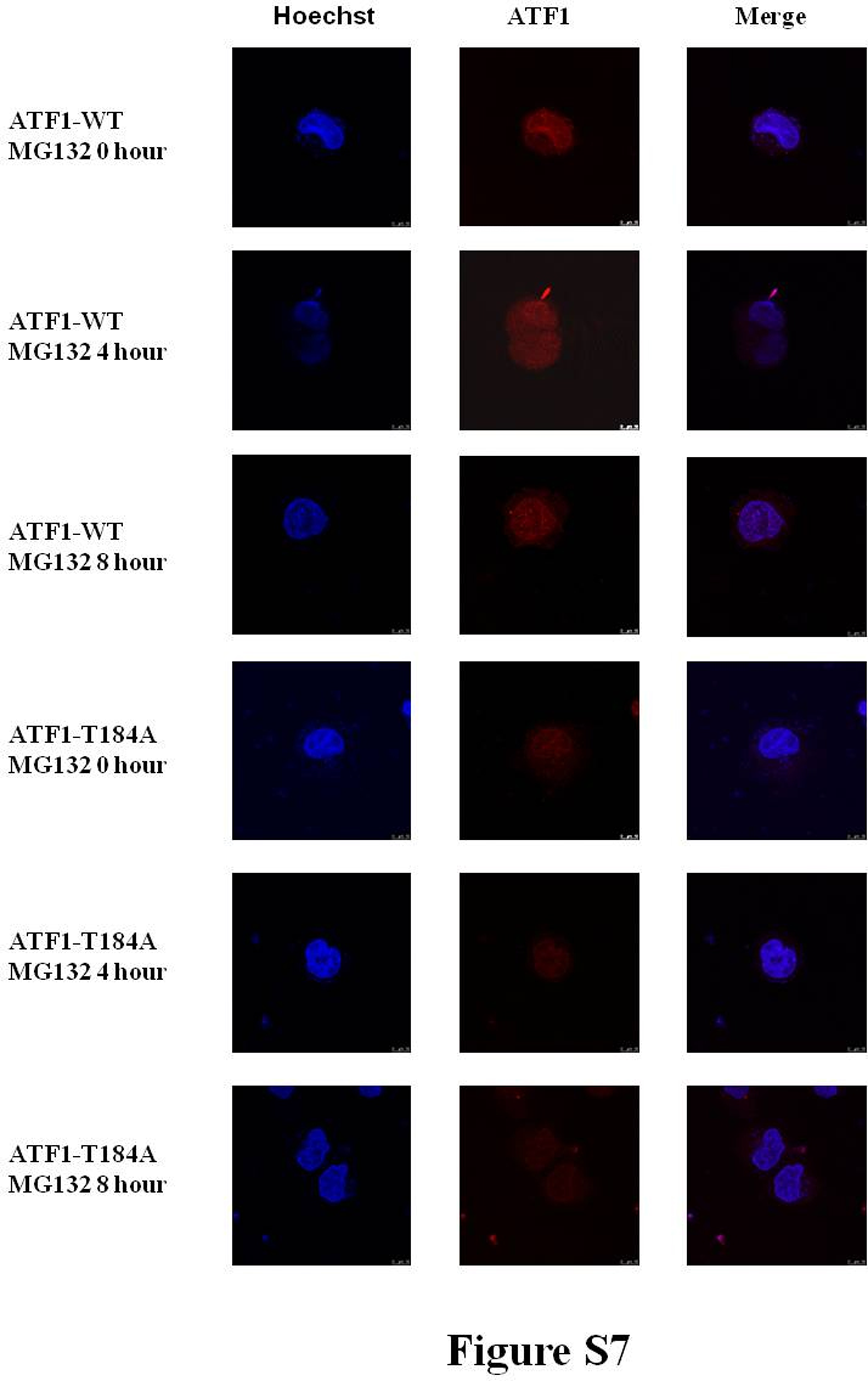

Supplement: Supplementary Figure 7 [file cddis2016349x8.tif]
